# Supplementary material for: Allelopathic Potential of Mangroves from the Red River Estuary against the Rice Weed Echinochloa crus-galli and Variation in Their Leaf Metabolome
Source: Plants (Basel). 2022 Sep 21;11(19):2464. doi: 10.3390/plants11192464 (PMC9573700; doi:10.3390/plants11192464)
Supplement: Supplementary file 1 [file plants-11-02464-s001.zip › plants-1839624-supplementary.pdf]

plants MDPI

Supplementary Material

Allelopathic Potential of Mangroves from the Red River Estuary against the Rice Weed *Echinochloa crus-galli* and Variation in Their Leaf Metabolome

Dounia Dhaou [1,*], Virginie Baldy [1], Dao Van Tan [2], Jean-Rémi Malachin [1], Nicolas Pouchard [1], Anaïs Roux [1], Sylvie Dupouyet [1], Stéphane Greff [1], Gérald Culioli [1], Thomas Michel [3], Catherine Fernandez [1] and Anne Bousquet-Mélou [1]

[1] IMBE, Aix Marseille University, Avignon University, CNRS, IRD, 13331 Marseille, France
[2] Department of Genetics-Biochemistry, Faculty of Biology, Hanoi National University of Education (HNUE), 131000 Hanoi, Vietnam
[3] Institut de Chimie de Nice, Université Côte d'Azur, CNRS, UMR 7272, 06108 Nice, France
* Correspondence: dounia.dhaou@imbe.fr

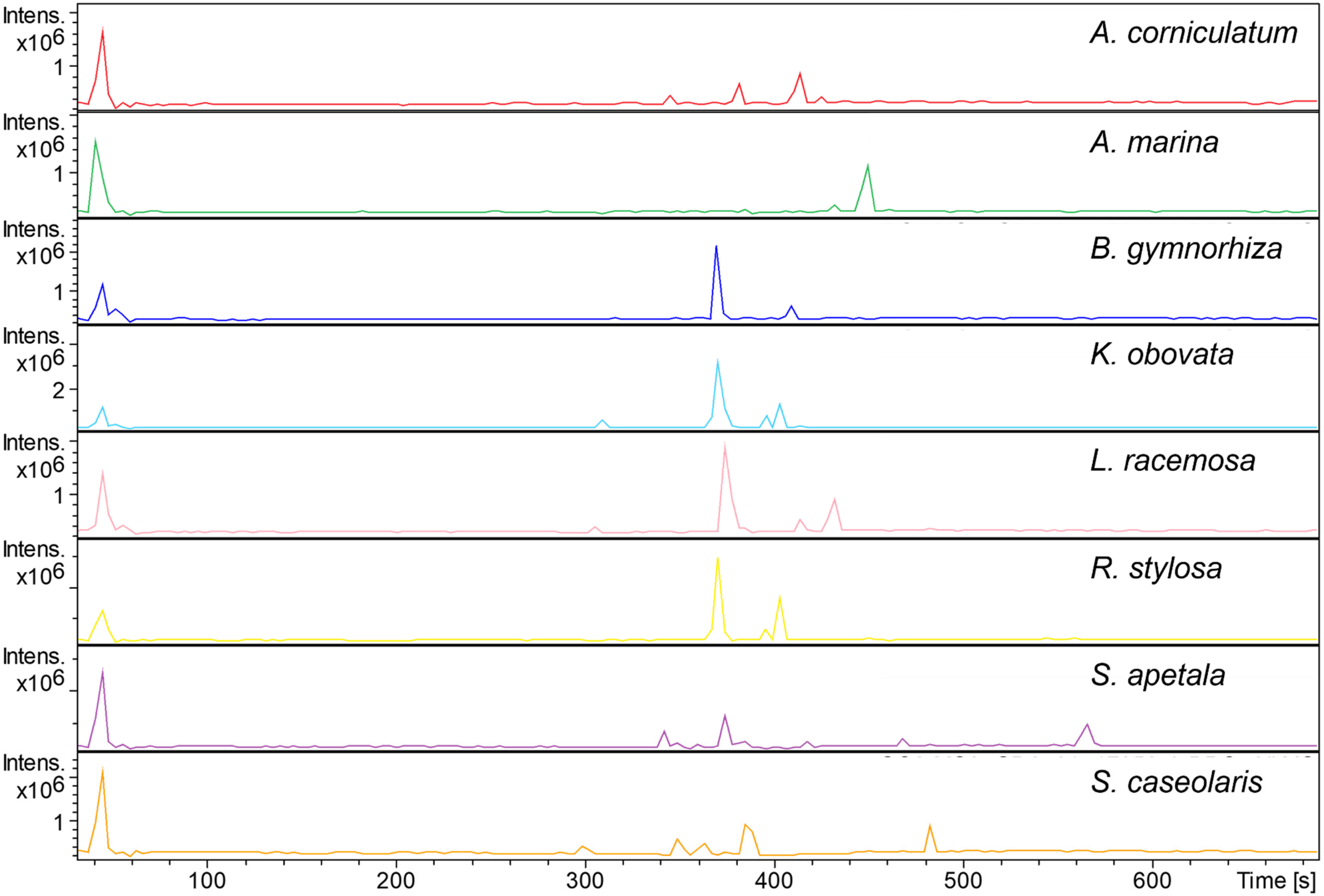

Figure S1. UHPLC-(-)ESI-qToF Base Peak Chromatograms (BPC) of leaf methanolic extracts from Aegiceras corniculatum, Avicennia marina, Bruguiera gymnorhiza, Kandelia obovata, Lumnitzera racemosa, Rhizophora stylosa, Sonneratia apetala and S. caseolaris.

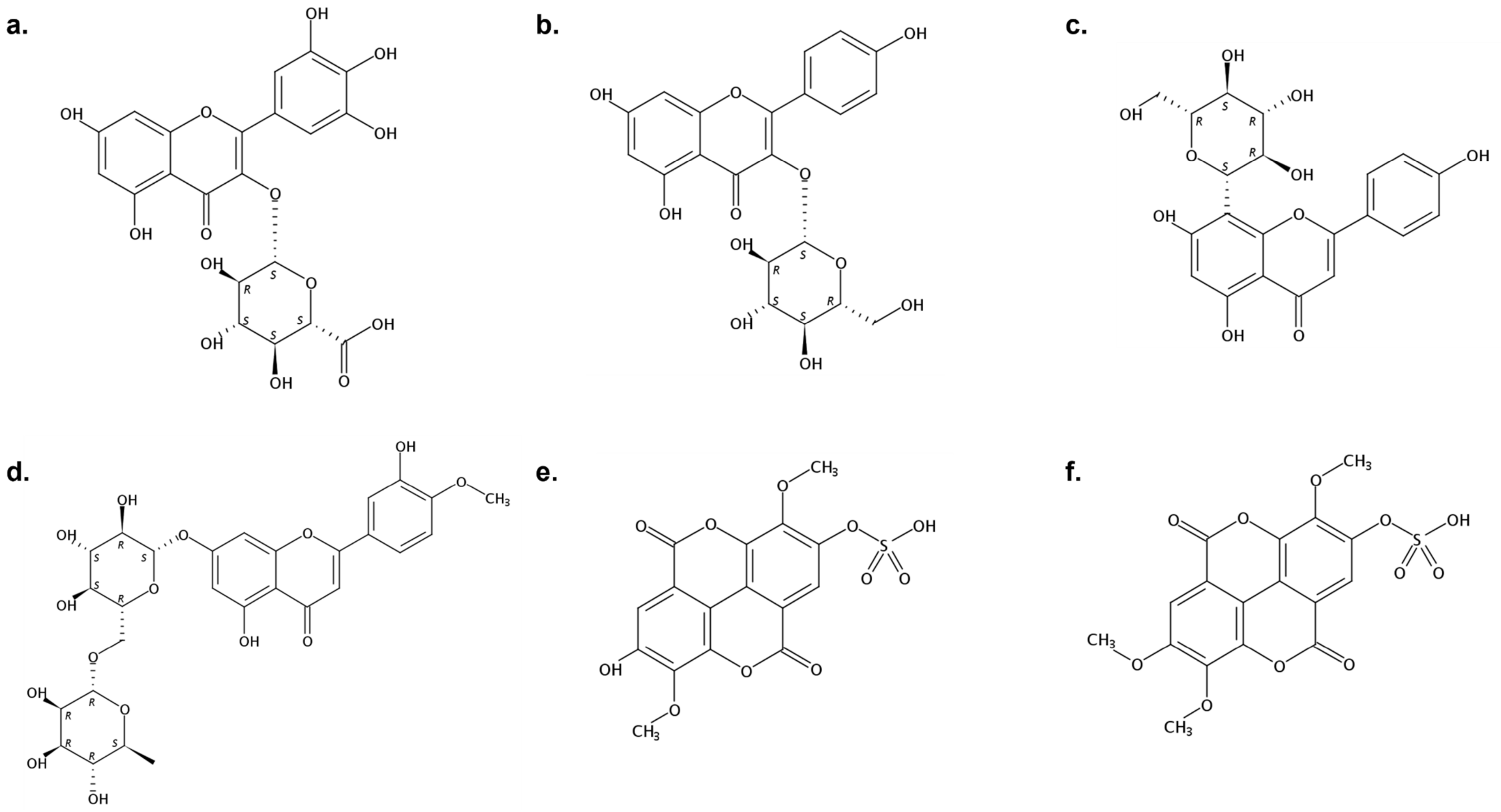

Figure S2. Chemical structures of some of the putatively annotated compounds: *Aegiceras corniculatum* compounds **Ac M1** (a, myricetin-3-glucuronide) and **Ac M3** (b, astragalin), and *Sonneratia apetala* compound **Sa M3** (c, vitexin), biomarker **M607T418 Sa M4 (**d**,** diosmin), compound **Sa M5** (e, dimethyl ellagic acid sulfate) and biomarker **M481T560 Sa M6** (f, 3,3′,4′-trimethyl ellagic acid 4-sulfate). All compounds and biomarkers were putatively annotated according to their HRMS/MS spectra.

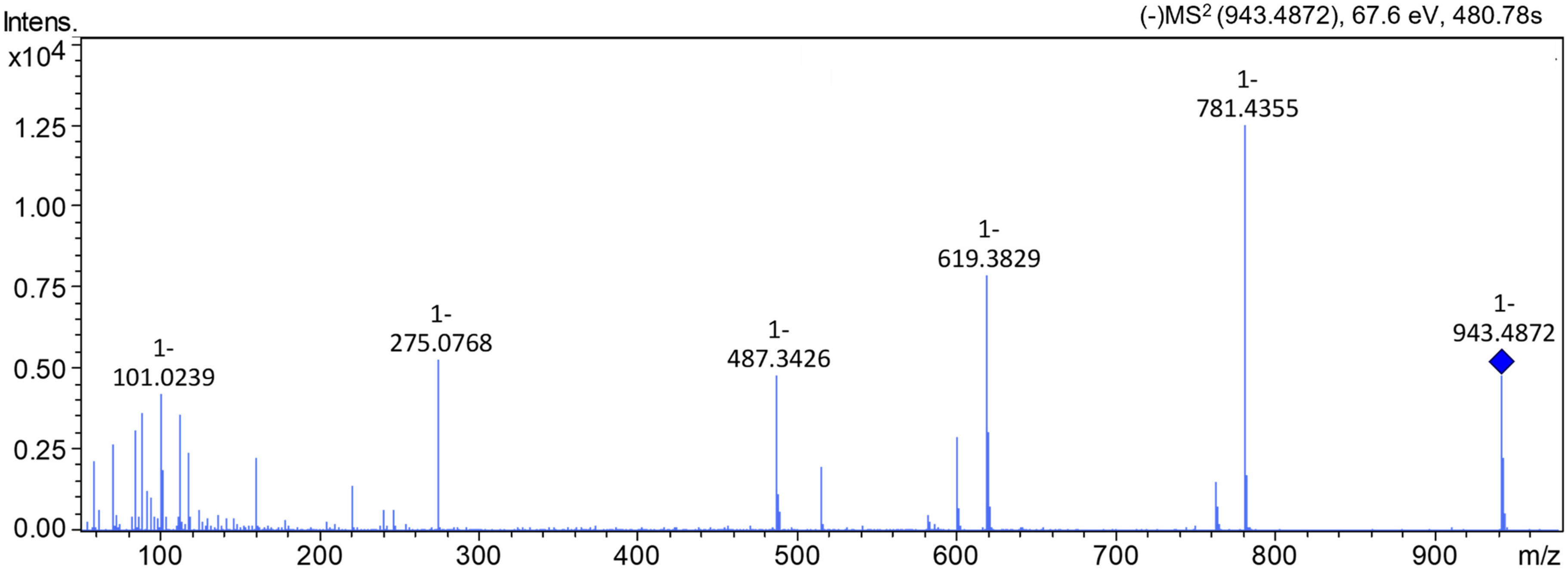

Figure S3. HRMS/MS spectrum of *Aegiceras corniculatum* biomarker M943T476 putatively annotated as (3β, 16α, 20α)-3,16,28-trihydroxyolean-12-en-29-oic acid 3-{O-β-D-glucopyranosyl (1→2)-O-[β-D-glucopyranosyl (1→4)]-α-L-arabinopyranoside}.

Detailed annotation

Biomarker M943T476 gave a [M-H]$^-$ at *m/z* 943.4872 and allowed to deduce the molecular formula $C_{47}H_{76}O_{19}$. MS/MS spectrum (Figure S3) was almost superposable to that of M781T518 (Figure S5) with the same hexose and pentose unit losses and same deduced aglycone fragment (putative triterpenic acid, $C_{30}H_{47}O_5$), except for an additional hexose loss. Based on MS spectra comparison in literature, this compound could be assigned as (3β,16α,20α)-3,16,28-trihydroxyolean-12-en-29-oic acid 3-{O-β-D-glucopyranosyl-(1→2)-O-[-D-glucopyranosyl-(1→4)]-α-L-arabinopyranoside}, a saponin already described in leaves of *A. corniculatum* collected in Vietnam [41].

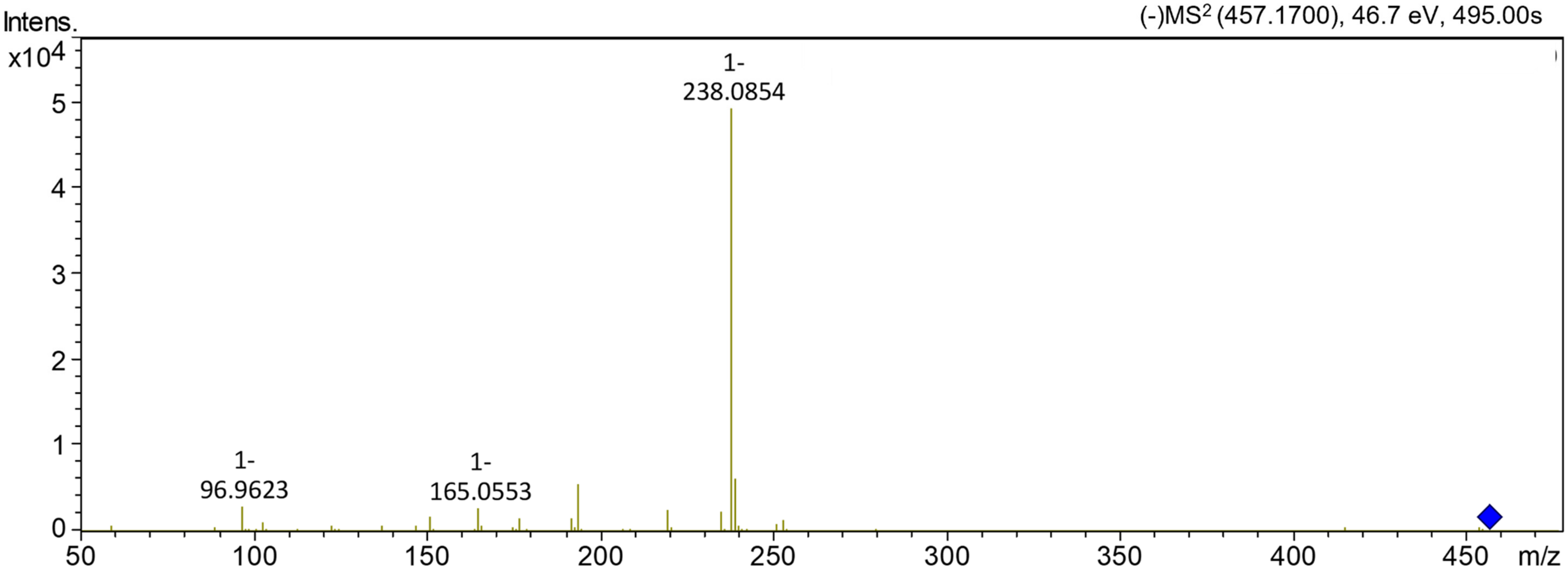

Figure S4. HRMS/MS spectrum of *Aegiceras corniculatum* unknown biomarker M457T495.

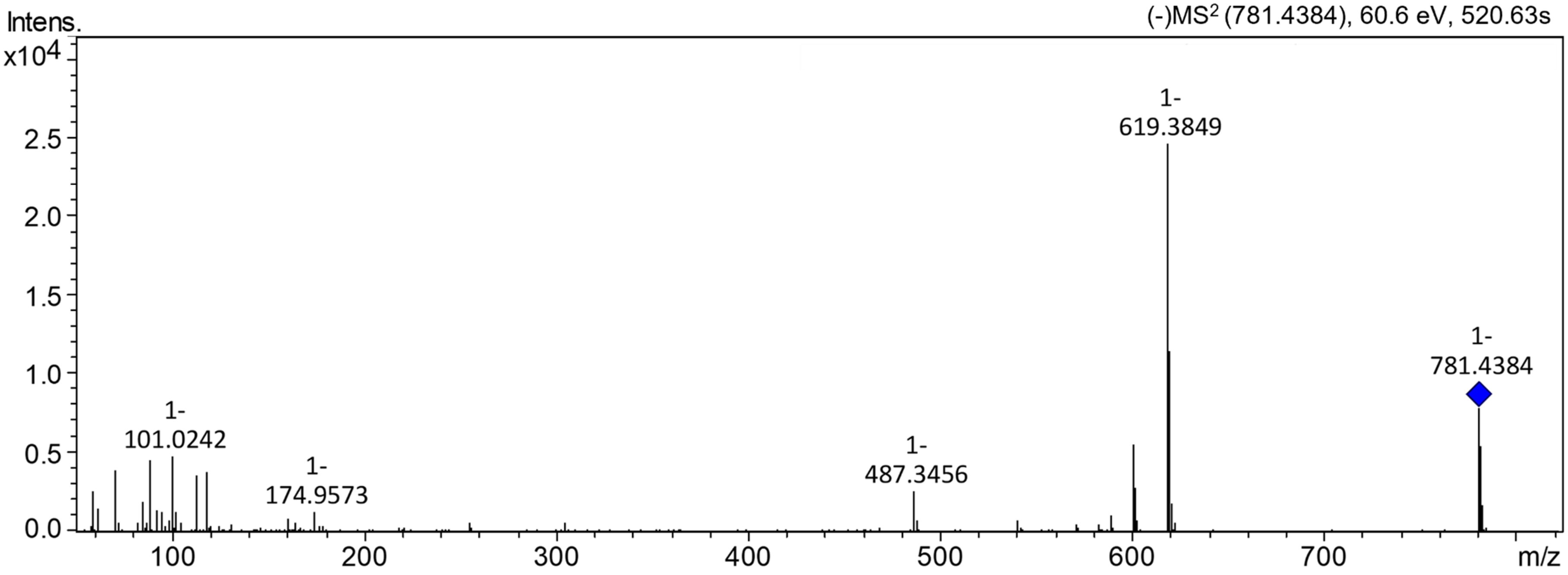

Figure S5. HRMS/MS spectrum of *Aegiceras corniculatum* biomarker M781T518 putatively annotated as a triterpenoid saponin.

Detailed annotation

Biomarker M781T518 specific to *A. corniculatum* leaves produced a deprotonated ion $[M-H]^-$ at *m/z* 781.4384 along with a chloride $[M+Cl]^-$ and a sodium formate $[M+HCOONa-H]^-$ adducts. MS/MS spectrum (Figure S5) showed the successive losses of a hexose $[M-H-162]^-$ and a pentose moiety $[M-H-162-132]^-$. The corresponding aglycone at *m/z* 487.3456 ($C_{30}H_{47}O_5$) could correspond to a triterpenic acid according to database interrogation. This compound was therefore putatively annotated as a triterpenoid saponin derivative.

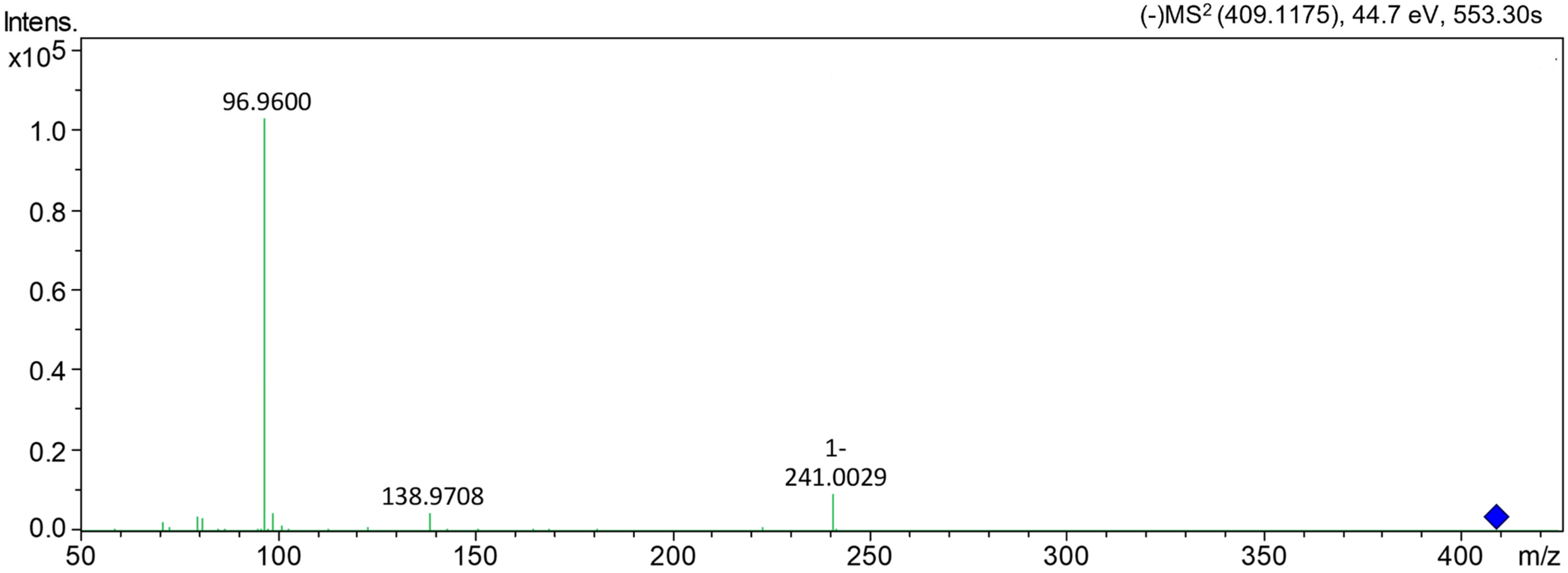

Figure S6. HRMS/MS spectrum of *Aegiceras corniculatum* biomarker **M410T546** putatively annotated as a **monoterpene sulfate.**

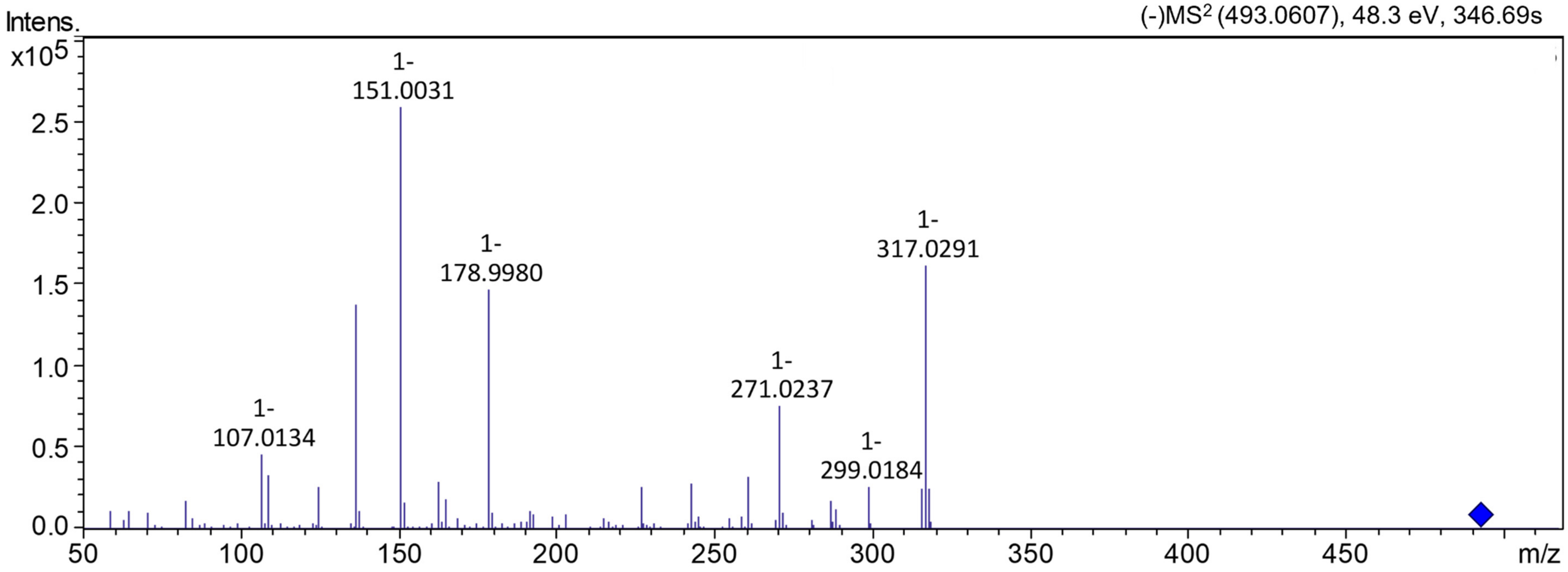

Figure S7. HRMS/MS spectrum of *Aegiceras corniculatum* major BPC compound **Ac M1** putatively annotated as **myricetin-3-glucuronide.**

Detailed annotation

BPC major compound Ac M1 presented a pseudo molecular ion $[M-H]^-$ at *m/z* 493.0607 corresponding to a raw formula of $C_{21}H_{17}O_{14}$. MS/MS fragment ion at *m/z* 317.0291 highlighted the loss of a glucuronic acid unit $[M-H-176]^-$ indicating a possible glucoronid flavonoid. Fragments at *m/z* 178.9980 and 151.0031 suggested the aglycone moiety as myricetin [43]. With a common fragmentation (*m/z* 317.0291 and 271.0237) described by De Rosso et al, 2015 [52], this compound might be myricetin-3-glucoronid (Figure S2a), found in leaves of mangrove *Conocarpus erectus* [42].

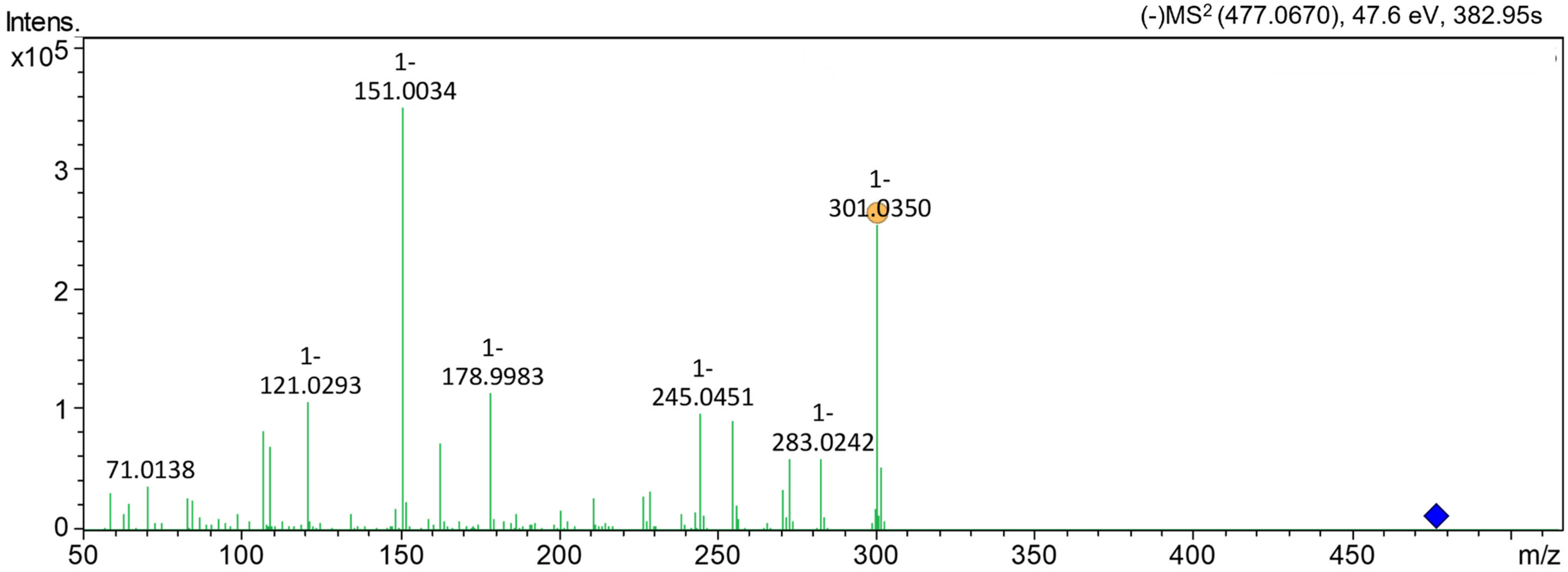

Figure S8. HRMS/MS spectrum of *Aegiceras corniculatum* major BPC compound **Ac M2** putatively annotated as **quercetin glucuronide** .

Detailed annotation

BPC major compound Ac M2 gave a [M-H]$^-$ at *m/z* 477.067 and showed a loss of glucuronic acid moiety as well with a fragment ion at *m/z* 301.0350 corresponding to the aglycone unit ($C_{15}H_9O_7$). According to literature, this metabolite could be a quercetin glucuronide already described in *Laguncularia racemosa* [43].

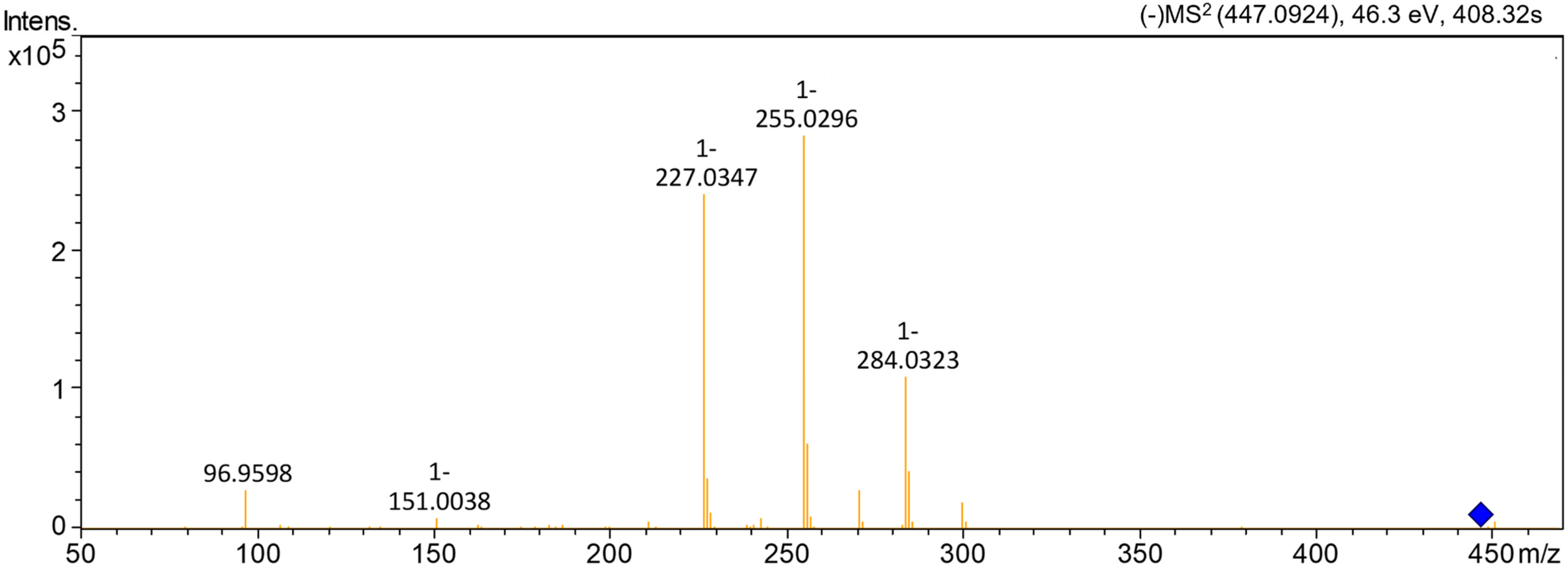

Figure S9. HRMS/MS spectrum of *Aegiceras corniculatum* major BPC compound Ac M3 putatively annotated as kaempferol-3-O-glucoside (astragalin)*. *Constructor statistical match factor (*m/z* defect and comparison of experimental vs theoretical isotopic patterns)

Detailed annotation

BPC major compound Ac M3 showed a pseudo molecular ion at *m/z* 447.0924. MS/MS spectrum matched with constructor database (Bruker db) compound kaempferol-3-glucoside (Figure S2b) also known as astragalin (common fragment ions *m/z* 284.0323, 255.0296 and 227.0347). Vinh et al, 2019 reported this metabolite in *A. corniculatum* leaves, supporting our assumptions [41].

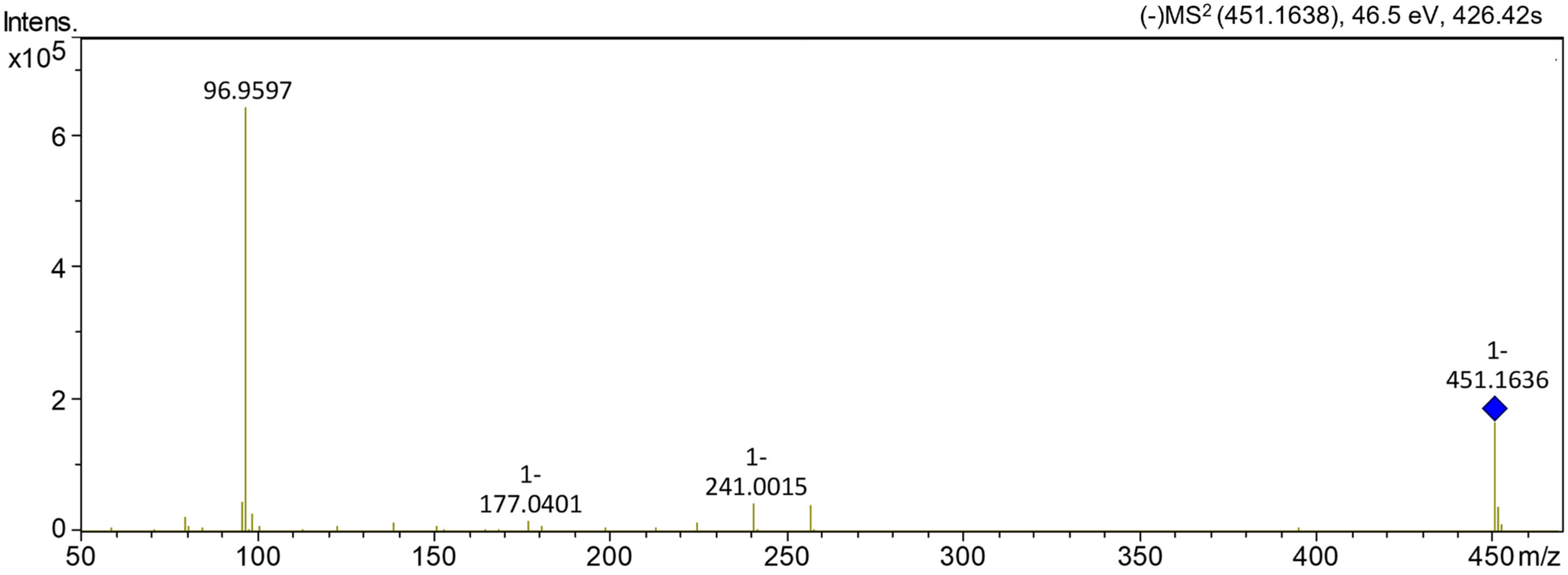

Figure S10. HRMS/MS spectrum of *Aegiceras corniculatum* **unknown** major BPC compound **Ac M4.**

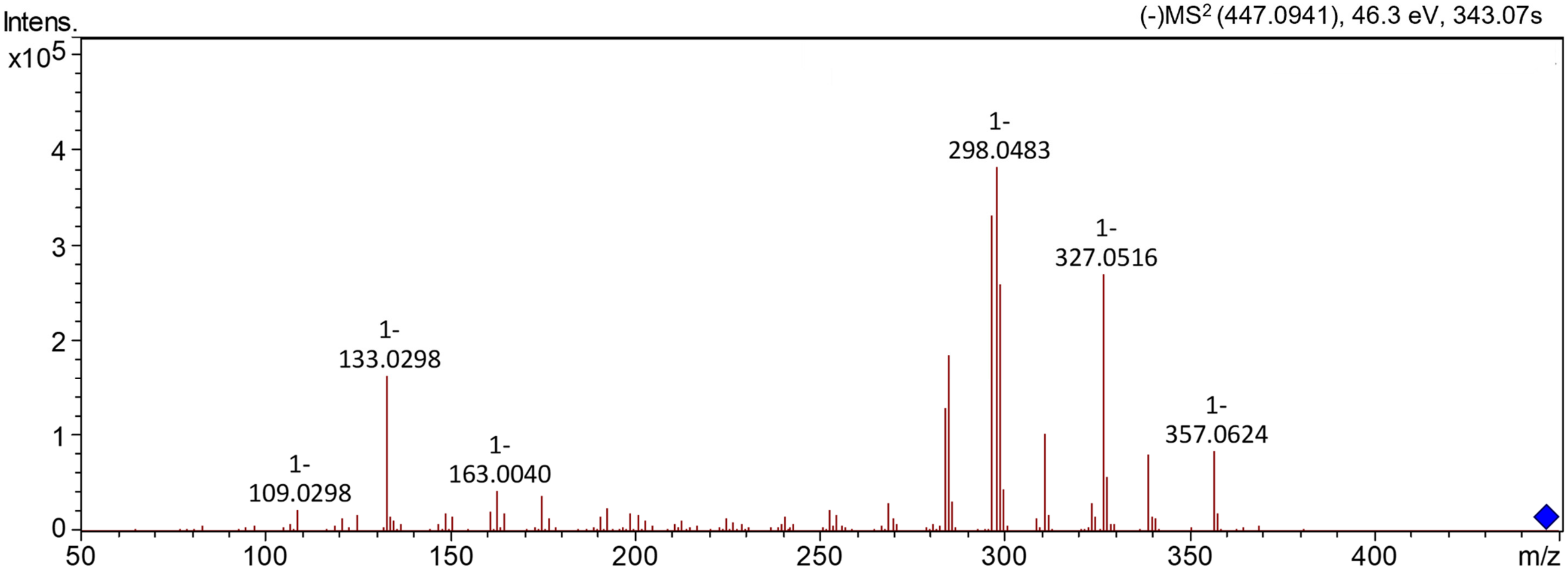

Figure S11. HRMS/MS spectrum of *Sonneratia apetala* major BPC compound **Sa M1** (biomarker **M895T343**) putatively annotated as **luteolin-7-O-β-glucoside.**

Detailed annotation

Biomarker M895T343 (also compound Sa M1) showed a deprotonated molecular ion $[M\text{-}H]^-$ at *m/z* 447.0941 consistent with a molecular formula of $C_{21}H_{20}O_{11}$. Precursor ion fragmentation led to the loss of a hexose moiety producing a deprotonated aglycone ion at *m/z* 285.0405 with a raw formula of $C_{15}H_9O_6$, which could correspond to luteolin or kaempferol. Characteristic fragment ions (*m/z* 199.0407, 175.0405, 151.0039 and 133.0298) observed in MS/MS spectrum [46] and luteolin already described in *S. apetala* fruits [47] allowed to suggest a luteolin glucoside. Although specifically more abundant in *S. apetala,* biomarker M895T343 was also detected in *S. caseolaris* and *B. gymnorhiza* extracts (Main text Figure 1b). For these additional reasons, this compound could be a luteolin 7-O-β-glucoside already described in *S. caseolaris* leaves and fruits [48,49].

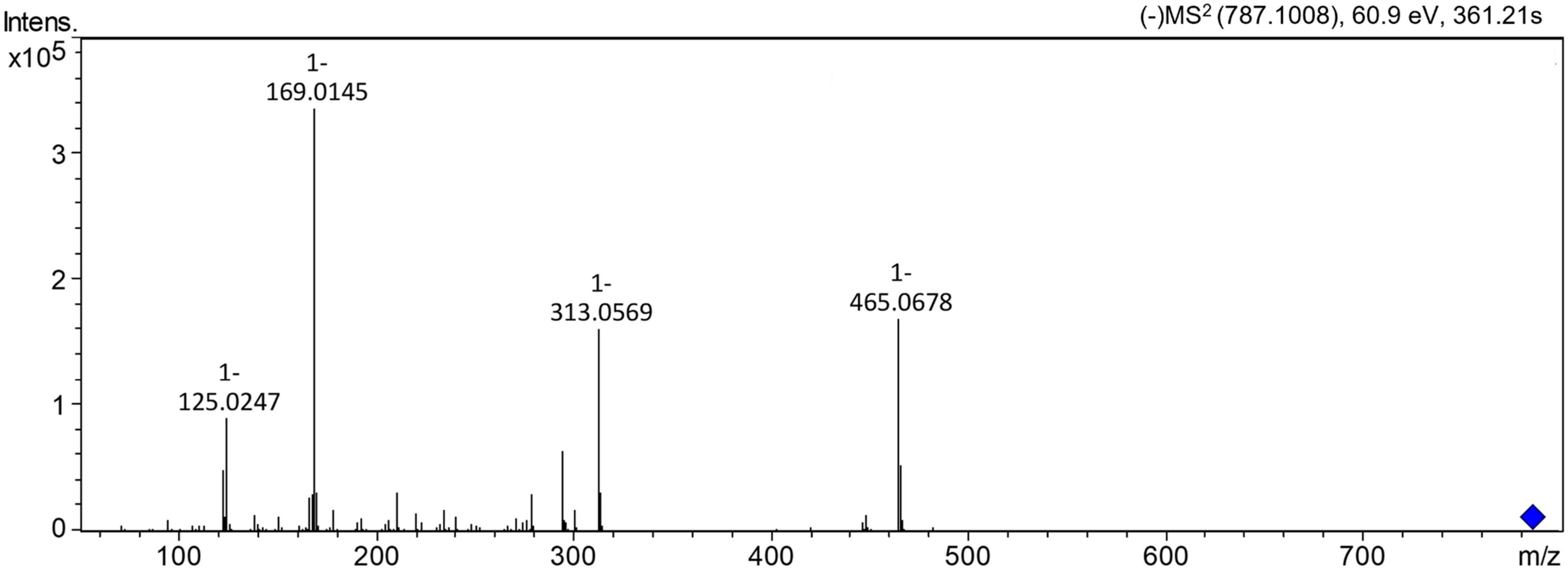

Figure S12. HRMS/MS spectrum of *Sonneratia apetala* major BPC compound **Sa M2** putatively annotated as **tetragalloyl glucose.**

Detailed annotation

Compound Sa M2 gave a deprotonated molecular ion $[M-H]^-$ at *m/z* 787.1008 which gave a raw formula of $C_{34}H_{27}O_{22}$. Online database interrogation led to matches with gallic acid derivatives. Characteristic fragment ions of tetragalloyl glucose [54] were detected at *m/z* 635.0915, 483.0789, 465.0678, 313.0569, 295.0463 and 169.0145 and showed successive galloyl moiety losses [M-H-152] [53]. This compound might be a 1,3,4,6-tetra-O-galloyl-β-D-glucose previously reported in the mangrove species *Excoecaria agallocha* [51].

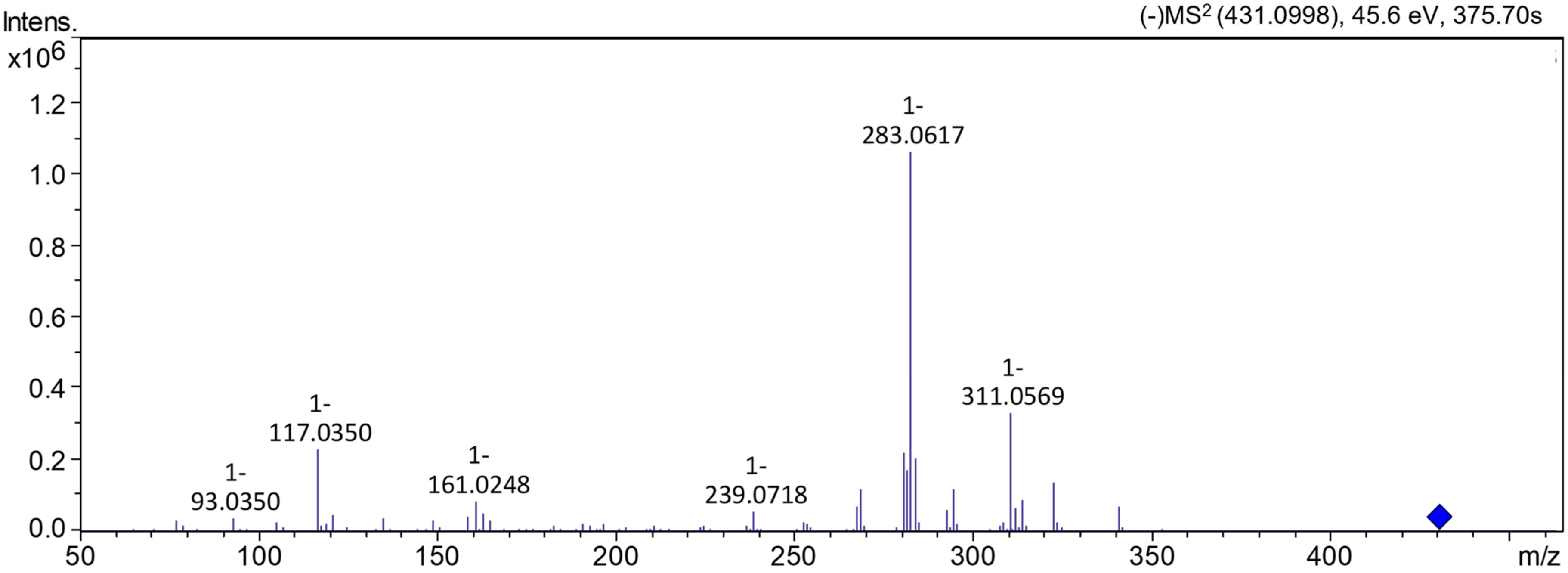

Figure S13. HRMS/MS spectrum of *Sonneratia apetala* major BPC compound **Sa M3** putatively annotated as **vitexin*.** * Constructor statistical match factor (*m/z* defect and comparison of experimental vs theoretical isotopic patterns)

Detailed annotation

Compound Sa M3 produced a deprotonated molecular ion at *m/z* 431.0998 allowing to deduce a molecular formula of $C_{21}H_{20}O_{10}$. MS/MS spectrum matched with those of vitexin (Figure S2c), a flavone glycoside, in the constructor database. Fragmentation showed two major product ions at *m/z* 311.0569 $[M-H-120]^-$ and 283.0617 common to the reference standard from database. This compound was already described in *S. apetala* leaves and branches collected in China [50].

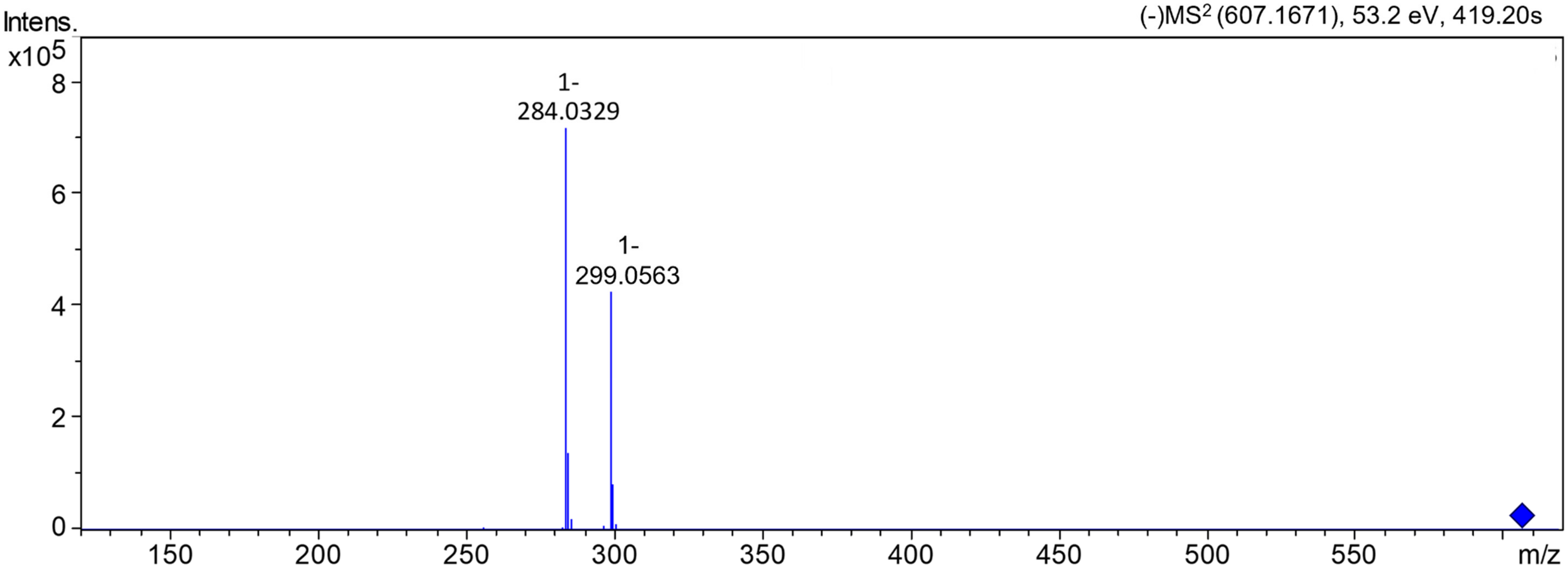

Figure S14. HRMS/MS spectrum of *Sonneratia apetala* major BPC compound **Sa M4** (biomarker **M607T418**) putatively annotated as **diosmin***.* Confirmed with commercial standards (in-house MS/MS spectra database)

Detailed annotation

Compound M607T418 (also compound Sa M4) gave a deprotonated molecular ion $[M-H]^-$ at *m/z* 607.1671 corresponding to the raw formula $C_{28}H_{32}O_{15}$. Interrogation of in-house database led to a match of MS/MS spectrum with diosmin (Figure S2d), a disaccharide derivative consisting of diosmetin substituted by a rutinose at position 7 via a glycosidic linkage. This biomarker broke down into two major product ions at *m/z* 299.0563 and 284.0329, confirming the characteristic loss of rutinose followed by a loss of a methyl. Finally, this assumption was further reinforced by the presence of diosmetin reported in two species from *Sonneratia* genus: *S. paracaseolaris* aerial parts [44] and *S. alba* leaves [45].

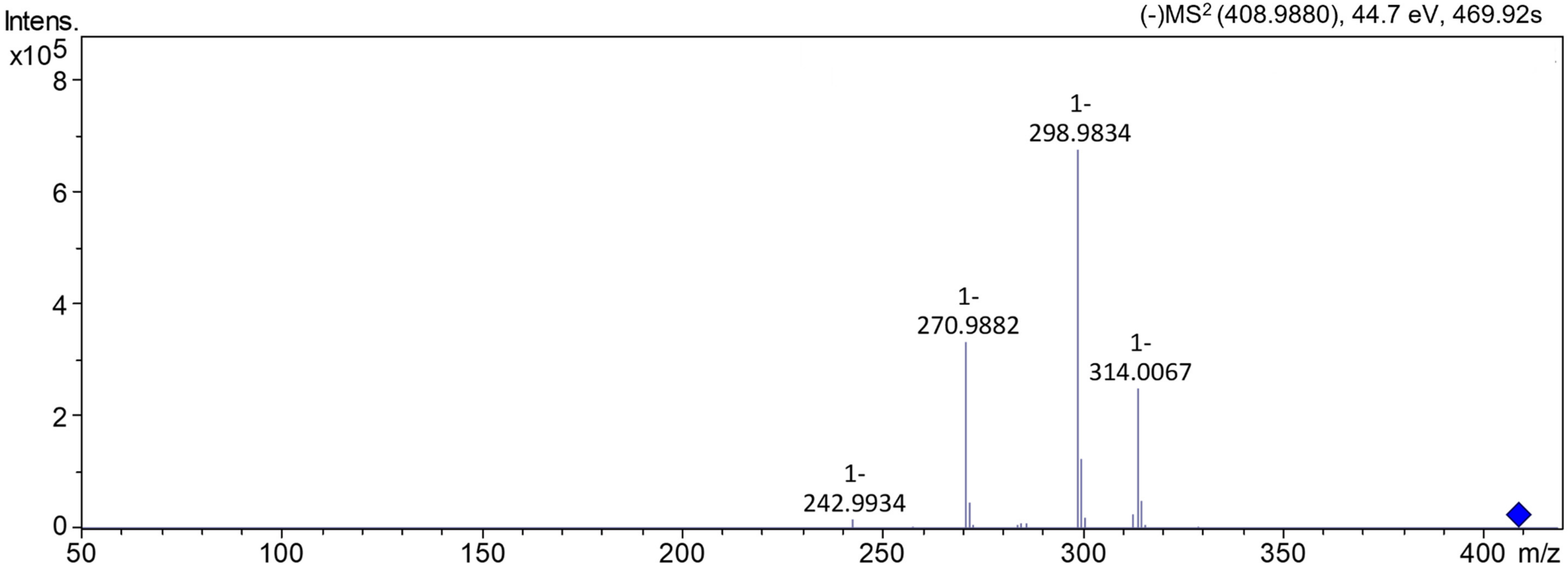

Figure S15. HRMS/MS spectrum of *Sonneratia apetala* major BPC compound **Sa M5** putatively annotated as **dimethyl ellagic acid sulfate.**

Detailed annotation

According to Manurung et al. 2021 [40], compound Sa M5 could be assigned to a dimethyl-ellagic acid sulfate (Figure S2e) since it followed the same fragmentation pattern as Sa M6 (Figure S16) (loss of a sulfate and methyl moieties) with a produced deprotonated molecular ion $[M-H]^-$ at *m/z* 408.9880 ($C_{16}H_{10}O_{11}S$).

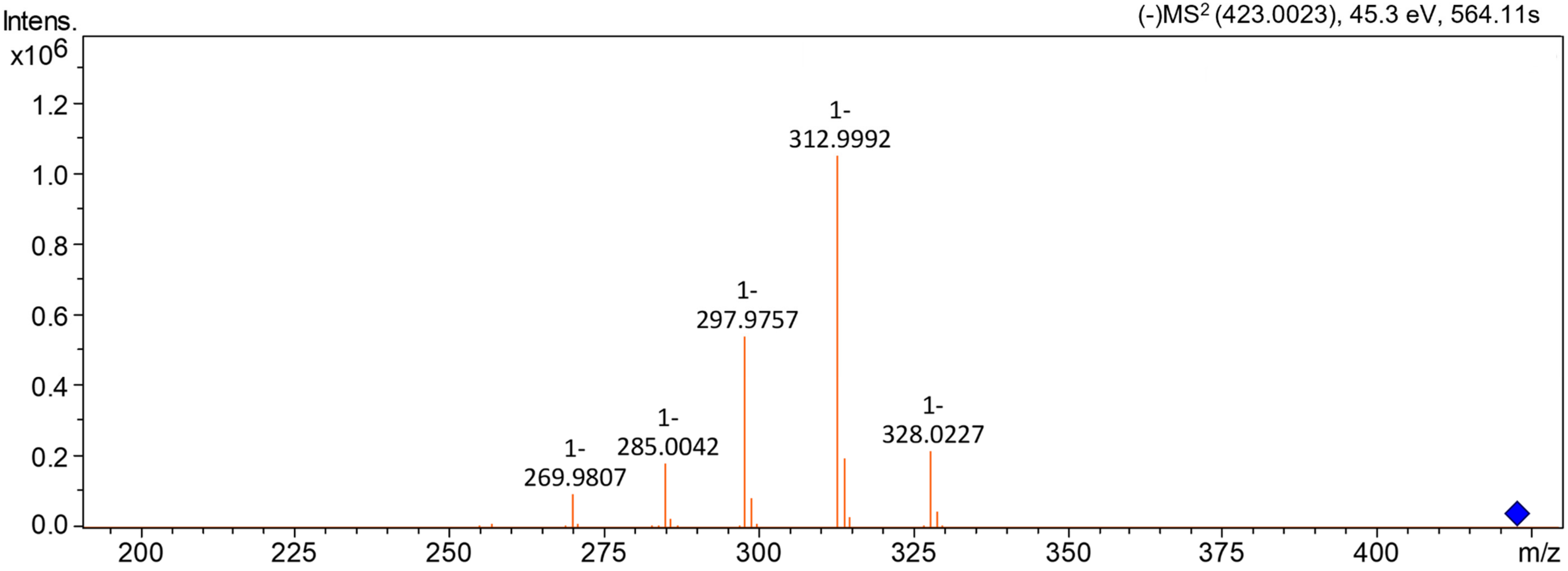

Figure S16. HRMS/MS spectrum of *Sonneratia apetala* major BPC compound **Sa M6** (biomarker **M481T560**) putatively annotated as **3,3′,4′-trimethylellagic acid 4-sulfate.**

Detailed annotation

Biomarker M481T560 (also BPC major compound Sa M6) of *S. apetala* leaves presented a deprotonated molecular ion $[M-H]^-$ at *m/z* 423.0023 with an isotopic pattern indicating the possible presence of a sulfur atom. The presence of a $[M-H-SO_3]^-$ fragment ion at *m/z* 343.0471 confirmed this assumption and suggested a sulfated metabolite with a raw formula of $C_{17}H_{12}SO_{11}$. Fragmentation pattern showed the successive loss of 3 methyl groups (*m/z* 328.0227, 312.9992, 297.9757). After comparison with literature, this compound was putatively annotated as 3,3',4′-tri-O-methyl-ellagic acid 4-sulfate (Figure S2f) which have recently been reported for the first time in mangrove species in the roots of *Lumnitzera littorea* and *L. racemosa* from Indonesia [40].

References

40. Manurung, J.; Kappen, J.; Schnitzler, J.; Frolov, A.; Wessjohann, L.A.; Agusta, A.; Muellner-Riehl, A.N.; Franke, K. Analysis of Unusual Sulfated Constituents and Anti-Infective Properties of Two Indonesian Mangroves, *Lumnitzera littorea* and *Lumnitzera racemos*a (Combretaceae). *Separations* **2021**, *8*, 82, doi:10.3390/separations8060082.
41. Vinh, L.B.; Nguyet, N.T.M.; Yang, S.Y.; Kim, J.H.; Thanh, N.V.; Cuong, N.X.; Nam, N.H.; Minh, C.V.; Hwang, I.; Kim, Y.H. Cytotoxic Triterpene Saponins from the Mangrove *Aegiceras corniculatum*. *Nat. Prod. Res.* **2019**, *33*, 628–634, doi:10.1080/14786419.2017.1402320.
42. Cruz, S.; Marroquín, M.N.; Cáceres, A. Biological Activity and Chemical Composition of Organic Extracts from Three Guatemalan Mangrove Trees. *Int. J. Phytocosmetics Nat. Ingred.* **2019**, *6*, 10–10, doi:10.15171/ijpni.2019.10.
43. Costa, F. das N.; Jerz, G.; Hewitson, P.; Figueiredo, F. de S.; Ignatova, S. *Laguncularia racemosa* Phenolics Profiling by Three-Phase Solvent System Step-Gradient Using High-Performance Countercurrent Chromatography with Off-Line Electrospray Mass-Spectrometry Detection. *Molecules* **2021**, *26*, 2284, doi:10.3390/molecules26082284.
44. Miao, S.; Man, Y.-Q.; Zhou, X.-L.; Yang, L.-J.; Gong, K.-K. Chemical Constituents from Mangrove Plant *Sonneratia paracaseolaris*. *Chin. Tradit. Herb. Drugs* **2018**, *49*, 1025–1030, doi:10.7501/j.issn.0253-2670.2018.05.006.
45. Sasmito, B.; Sulistiyati, T.D.; Hardoko, H. Phytochemicals and Identification of Antioxidant Compounds from Ethanol Extract of *Sonneratia alba* Leaves and Bark. *Russ. J. Agric. Socio-Econ. Sci.* **2019**, *95*, 190–196, doi:10.18551/rjoas.2019-11.26.
46. Li, Z.-H.; Guo, H.; Xu, W.-B.; Ge, J.; Li, X.; Alimu, M.; He, D.-J. Rapid Identification of Flavonoid Constituents Directly from PTP1B Inhibitive Extract of Raspberry (*Rubus idaeus* L.) Leaves by HPLC–ESI–QTOF–MS-MS. *J. Chromatogr. Sci.* **2016**, *54*, 805–810, doi:10.1093/chromsci/bmw016.
47. Yi, X.; Jiang, S.; Qin, M.; Liu, K.; Cao, P.; Chen, S.; Deng, J.; Gao, C. Compounds from the Fruits of Mangrove *Sonneratia apetala*: Isolation, Molecular Docking and Antiaging Effects Using a *Caenorhabditis elegans* Model. *Bioorganic Chem.* **2020**, *99*, 103813, doi:10.1016/j.bioorg.2020.103813.
48. Wu, S.-B.; Wen, Y.; Li, X.-W.; Zhao, Y.; Zhao, Z.; Hu, J.-F. Chemical Constituents from the Fruits of *Sonneratia caseolaris* and *Sonneratia ovata* (Sonneratiaceae). *Biochem. Syst. Ecol.* **2009**, *37*, 1–5, doi:10.1016/j.bse.2009.01.002.
49. Sadhu, S.K.; Ahmed, F.; Ohtsuki, T.; Ishibashi, M. Flavonoids from *Sonneratia caseolaris*. *J. Nat. Med.* **2006**, *60*, 264–265, doi:10.1007/s11418-006-0029-3.
50. Wu, Y.-L.; Chen, J.-F.; Jiang, L.-Y.; Wu, X.-L.; Liu, Y.-H.; Gao, C.-J.; Wu, Y.; Yi, X.-Q.; Su, Z.-R.; Cai, J.; et al. The Extract of *Sonneratia apetala* Leaves and Branches Ameliorates Hyperuricemia in Mice by Regulating Renal Uric Acid Transporters and Suppressing the Activation of the JAK/STAT Signaling Pathway. *Front. Pharmacol.* **2021**, *12*, 1826, doi:10.3389/fphar.2021.698219.
51. Li, Y.; Yu, S.; Liu, D.; Proksch, P.; Lin, W. Inhibitory Effects of Polyphenols toward HCV from the Mangrove Plant *Excoecaria agallocha* L. *Bioorg. Med. Chem. Lett.* **2012**, *22*, 1099–1102, doi:10.1016/j.bmcl.2011.11.109.
52. De Rosso, M.; Panighel, A.; Vedova, A.; Gardiman, M.; Flamini, R. Characterization of Non-Anthocyanic Flavonoids in Some Hybrid Red Grape Extracts Potentially Interesting for Industrial Uses. *Molecules* **2015**, *20*, 18095–18106, doi:10.3390/molecules201018095.
53. Saldanha, L.L.; Vilegas, W.; Dokkedal, A.L. Characterization of Flavonoids and Phenolic Acids in *Myrcia bella* Cambess. Using FIA-ESI-IT-MS(n) and HPLC-PAD-ESI-IT-MS Combined with NMR. *Mol. Basel Switz.* **2013**, *18*, 8402–8416, doi:10.3390/molecules18078402.
54. Singh, A.; Bajpai, V.; Kumar, S.; Sharma, K.R.; Kumara, B. Profiling of Gallic and Ellagic Acid Derivatives in Different Plant Parts of *Terminalia arjuna* by HPLC-ESI-QTOF-MS/MS. *Nat. Prod. Commun.* **2016**, *11*, 239–244.
